# USP12 promotes breast cancer angiogenesis by maintaining midkine stability

**DOI:** 10.1038/s41419-021-04102-y

**Published:** 2021-11-11

**Authors:** Bin Sheng, Zichao Wei, Xiaowei Wu, Yi Li, Zhihua Liu

**Affiliations:** State Key Laboratory of Molecular Oncology, National Cancer Center/National Clinical Research Center for Cancer/Cancer Hospital, Chinese Academy of Medical Sciences and Peking Union Medical College, Beijing, 100021 PR China

**Keywords:** Breast cancer, Breast cancer

## Abstract

Deubiquitinases (DUBs) have important biological functions, but their roles in breast cancer metastasis are not completely clear. In this study, through screening a series of DUBs related to breast cancer distant metastasis-free survival (DMFS) in the Kaplan-Meier Plotter database, we identified ubiquitin-specific protease 12 (USP12) as a key deubiquitinating enzyme for breast cancer metastasis. We confirmed this via an orthotopic mouse lung metastasis model. We revealed that the DMFS of breast cancer patients with high USP12 was worse than that of others. Knockdown of USP12 decreased the lung metastasis ability of 4T1 cells, while USP12 overexpression increased the lung metastasis ability of these cells in vivo. Furthermore, our results showed that the supernatant from USP12-overexpressing breast cancer cells could promote angiogenesis according to human umbilical vein endothelial cell (HUVEC) migration and tube formation assays. Subsequently, we identified midkine (MDK) as one of its substrates. USP12 could directly interact with MDK, decrease its polyubiquitination and increase its protein stability in cells. Overexpression of MDK rescued the loss of angiogenesis ability mediated by knockdown of USP12 in breast cancer cells in vitro and in vivo. There was a strong positive relationship between USP12 and MDK protein expression in clinical breast cancer samples. Consistent with the pattern for USP12, high MDK expression predicted lower DMFS and overall survival (OS) in breast cancer. Collectively, our study identified that USP12 is responsible for deubiquitinating and stabilizing MDK and leads to metastasis by promoting angiogenesis. Therefore, the USP12–MDK axis could serve as a potential target for the therapeutic treatment of breast cancer metastasis.

## Introduction

Breast cancer is the most common malignant tumour in the world [1]. In breast cancer patients, it is not the primary tumour but its metastases at distant sites that are the main cause of death [2]. Because of the lack of effective therapeutic targets for breast cancer metastases to date, it is necessary and urgent for us to find a novel treatment target for breast cancer metastasis.

Tumour metastasis is a complex multistep process involving changes in multidimensional analytical network systems such as genomes, transcription groups, proteomics and metabolites. The post-translational modification, ubiquitination and deubiquitination of proteins can cause changes in the molecular network related to tumour metastasis by regulating protein function or degradation. Deubiquitination mediated by many deubiquitinases (DUBs) regulates the level of substrate proteins by cleaving ubiquitin chains and participates in a variety of cellular processes [3, 4]. DUBs play a role in tumour metastasis progression [5], including degradation of extracellular matrix [6], epithelial–mesenchymal transition [7], angiogenesis [8, 9], circulating tumour cell behaviour [10], anoikis resistance [11] and so on. Proteasome and ubiquitin E3 ligase inhibitors and first-generation DUB inhibitors are now approaching clinical trials [12]. Although DUBs have an important role in the process of tumour formation and have bright prospects for tumour treatment, the role and mechanisms of DUBs in the process of breast cancer metastasis are not fully understood.

To investigate the role and mechanisms of DUBs in breast cancer metastasis, more than ten DUBs related to breast cancer DMFS were screened from the Kaplan-Meier (KM) Plotter database. Then, we further verified these DUBs in the orthotopic mouse lung metastasis model and identified that ubiquitin-specific protease 12 (USP12) is responsible for breast cancer metastasis. USP12 is a member of the USP family with deubiquitinating activity [13]. It has been reported that USP12 is involved in the development of cancer, including prostate cancer and other cancers [14–17]. However, the association of USP12 with metastasis still lacks research-based evidence.

In this study, we found that USP12 accelerates breast cancer metastasis by promoting angiogenesis, which had not been previously described. It promotes angiogenesis by deubiquitinating and stabilizing the midkine (MDK) protein. MDK is a heparin-binding growth factor that is expressed at abnormally high levels in various cancers [18], especially during tumour progression into more advanced stages [19]. It plays a key role in the acquisition of critical hallmarks of cancer, including cell growth, metastasis and angiogenesis [20]. Our results demonstrated that USP12 directly binds and deubiquitinate MDK, leading to the upregulation of MDK and the promotion of angiogenesis and metastasis, which suggests that USP12 is a key mediator of breast cancer metastasis and provides us with a possible opportunity for therapy against metastasis.

## Results

### Identification of USP12 as a key molecule in breast cancer metastasis

**Fig. 1:**
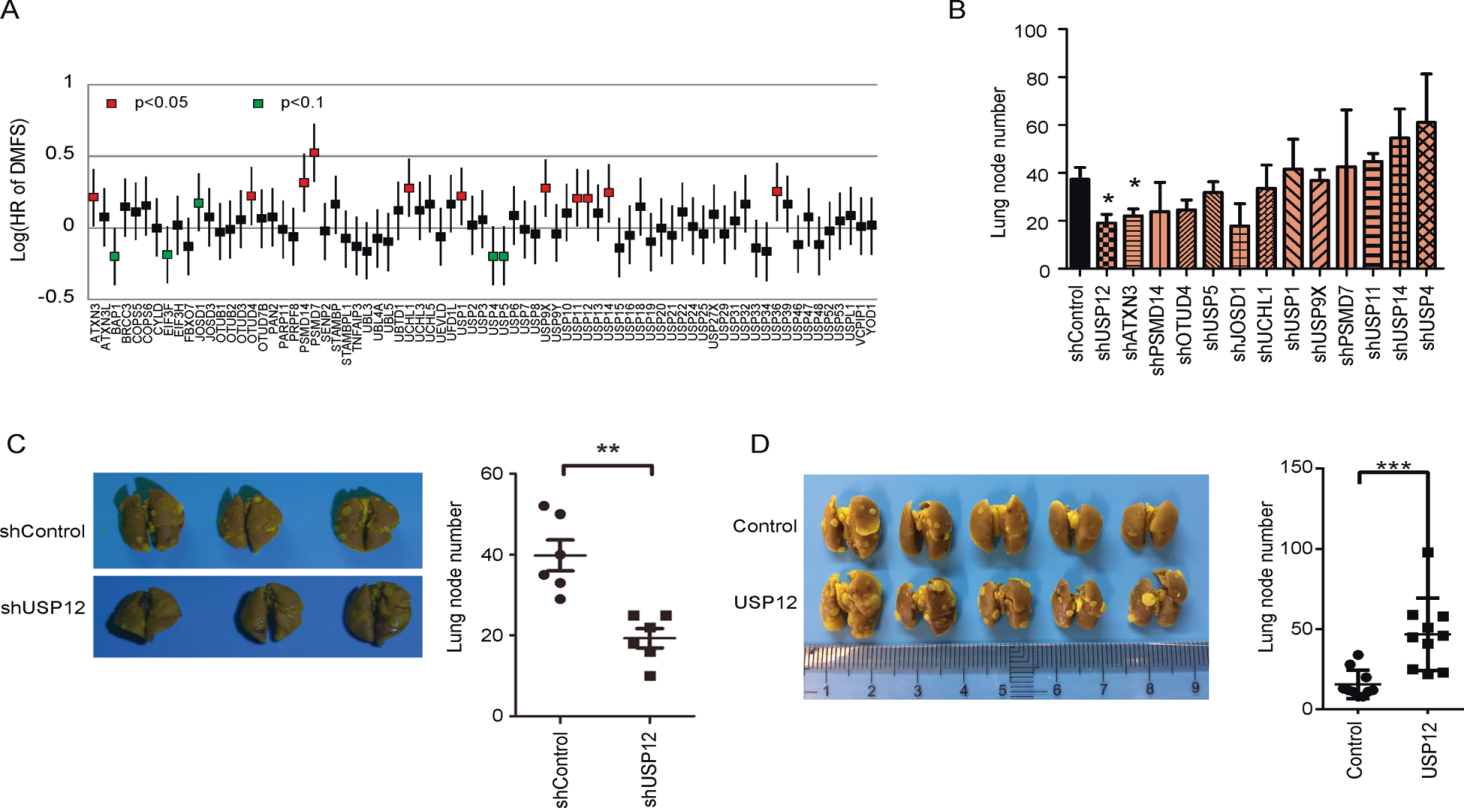
USP12 is a key DUB of breast cancer metastasis. **A** The relationships between 74 DUBs (the other 33 DUBs had no data in the database) and breast cancer DMFS were analysed in the KM Plotter database (http://kmplot.com/analysis/). **B** Statistics related to the number of lung metastasis nodules after the injection of DUB knockdown and control 4T1 cells into mouse mammary pads for 5 weeks are shown, *p < 0.05. **C** Stable USP12 knockdown and control 4T1 cells were injected into the mammary pads of 5–6-week-old BALB/c mice, and the lungs were harvested for picric acid staining. Representative images (left panel) and quantitative results (right panel) are shown. **D** Stable USP12-overexpression and control 4T1 cells were injected into the mammary pads of 5–6-week-old BALB/c mice. Lung metastasis was evaluated at week 4 after injection. Representative images (left panel) and quantitative results (right panel) are shown; the data are presented as the mean ± SD; ^*^p < 0.05, ^**^p < 0.01 ^***^p < 0.001.

We analysed the relationship between 74 DUBs (the other 33 DUBs had no data in the database) and the distant metastasis-free survival (DMFS) of breast cancer patients through the KM Plotter database (http://kmplot.com/analysis/). A total of 11 DUBs (*p* < 0.05) were significantly associated with breast cancer DMFS (Fig. 1A). For further confirmation, the DUBs significantly associated with DMFS and several DUBs marginally associated with DMFS (*p* < 0.1) were validated using an orthotopic mouse lung metastasis model (the knockdown efficiency of shRNA was showed in Fig. S1). We found that knocking down USP12 had the most significant inhibitory effect on the lung metastasis of 4T1 cells (Fig. 1B, C). Furthermore, the results in the in vivo mouse model showed that 4T1-overexpressing USP12 cells exhibited higher lung metastasis potential than control cells (Fig. 1D). Therefore, USP12 was identified as a key molecule responsible for breast cancer metastasis.

### USP12 promotes breast cancer angiogenesis

**Fig. 2:**
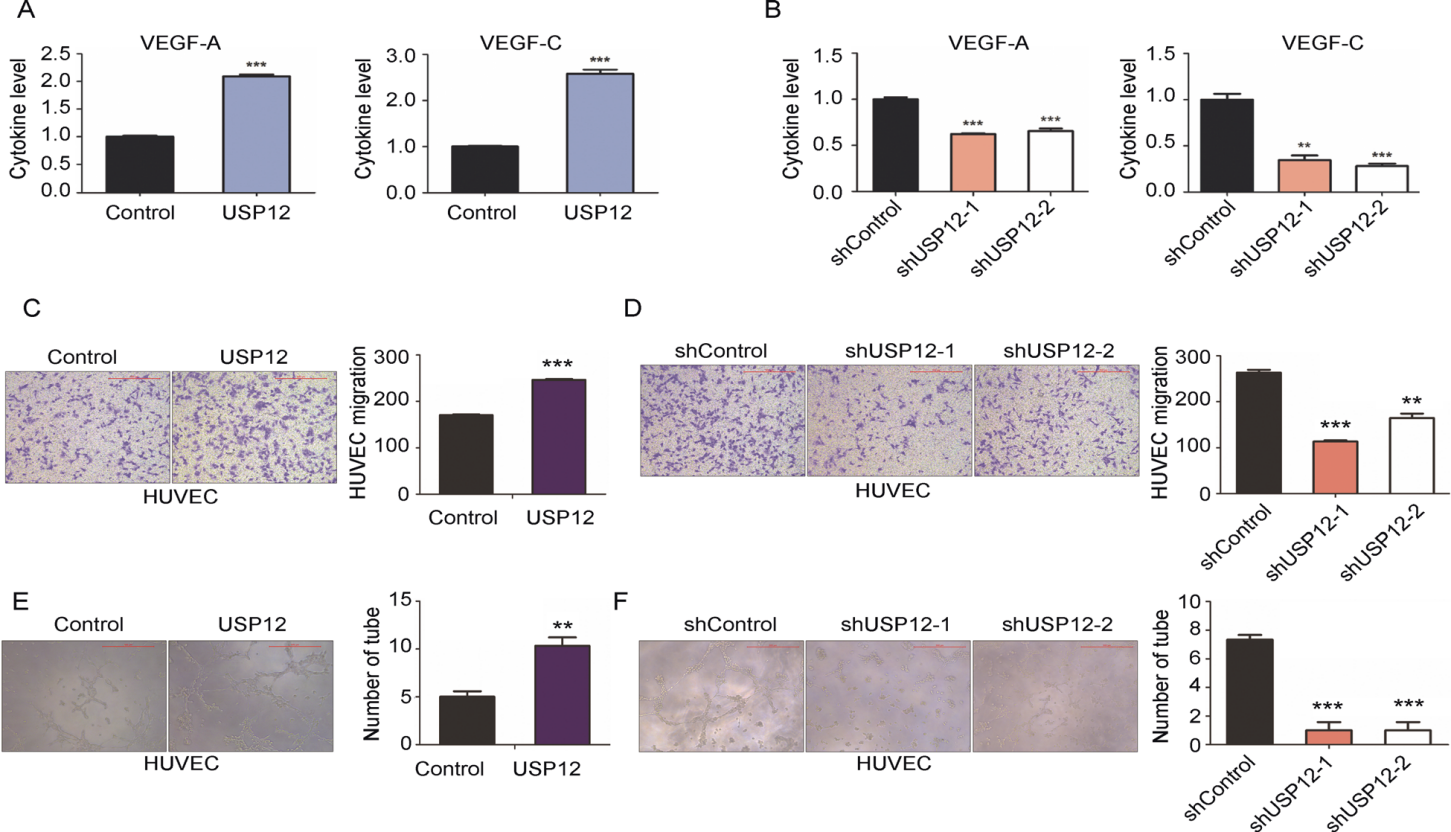
USP12-induced angiogenesis in breast cancer. **A** The relative expression of VEGF-A and VEGF-C in the supernatant of MDA-MB-231 cells overexpressing USP12 was detected by ELISA. **B** The relative expression of VEGF-A and VEGF-C in the supernatant of MDA-MB-231 cells with USP12 knockdown was detected by ELISA. **C**, **D** Migration of HUVECs treated with supernatant from MDA-MB-231 cells with USP12 overexpression (**C**) and USP12 knockdown (**D**). Representative images are shown in the left panel. The quantitative results are shown in the right panel. **E**, **F**. Tube formation assay of HUVECs treated with supernatant from MDA-MB-231 cells with USP12 overexpression (**E**) and USP12 knockdown (**F**). Representative images are shown in the left panel. The quantitative results are shown in the right panel. The experiments were repeated three times. ^**^p < 0.01, ^***^p < 0.001; mean ± SD.

To clarify the mechanism by which USP12 promotes metastasis, we first assessed the migration and invasion of MDA-MB-231 cells after knockdown and overexpression of USP12. However, neither overexpression (Fig. S2A) nor knockdown of USP12 (Fig. S2B) affected the migration and invasion abilities of MDA-MB-231 cells, as demonstrated by Transwell migration and invasion assays in vitro. Then, we detected angiogenesis-related factors in the cell supernatant and found that overexpression of USP12 promoted the secretion of VEGF-A and VEGF-C (Fig. 2A), and knockdown of USP12 decreased the secretion of these factors (Fig. 2B) in MDA-MB-231 cells. To further clarify the mechanism by which USP12 increased metastasis by promoting angiogenesis, we performed human umbilical vein endothelial cell (HUVEC) migration and tube formation assays in vitro. Our results showed that supernatant from USP12-overexpressing MDA-MB-231 and MCF7 cells promoted the migration of HUVECs (Figs. 2C and S2C). In contrast, treatment with supernatant from USP12-knockdown MDA-MB-231 and MCF7 cells decreased HUVEC migration (Figs. 2D and S2D). In addition, the tube formation assay results showed that compared with the supernatant from control cells, the supernatant from USP12-overexpressing MDA-MB-231 and MCF7 cells remarkably promoted HUVEC tube formation (Figs. 2E and S2C), and the supernatant from USP12-knockdown MDA-MB-231 and MCF7 cells suppressed tube formation of HUVECs (Figs. 2F and S2D).

### USP12 directly targets MDK

**Fig. 3:**
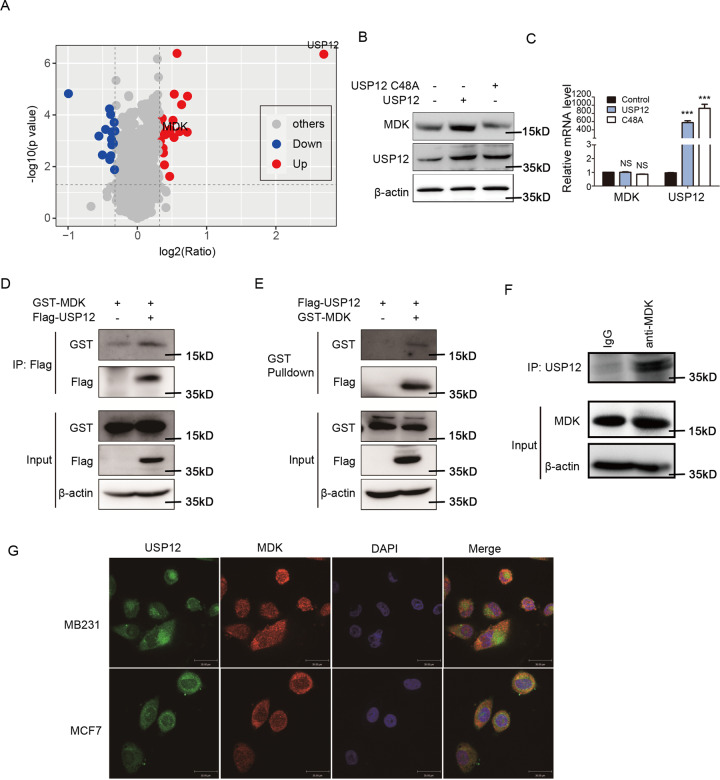
MDK is a substrate of USP12. **A** The quantitative proteomics of USP12-overexpressing and control HEK293T cells were analysed using the iTRAQ technique, and 36 DEPs (including 21 upregulated proteins and 15 downregulated proteins) were identified. **B**, **C** USP12, USP12 mutant (C48A) and control vectors were transfected into HEK293T cells, and the protein and mRNA levels of MDK and USP12 were detected by western blotting (**B**) and RT-qPCR (**C**). **D** USP12-Flag and the MDK-GST plasmid were co-transfected into HEK293T cells, and USP12-Flag was immunoprecipitated with anti-Flag antibody. **E** USP12-Flag and the MDK-GST plasmid were co-transfected into HEK293T cells, and MDK-GST was pulled down with glutathione-Sepharose 4B slurry beads. **F** Endogenous USP12 was captured by anti-MDK antibody from MDA-MB-231 cells, and the endogenous USP12 and MDK were examined by immunoblotting. **G** Endogenous USP12 and MDK expression in MDA-MB-231 (upper) and MCF7 (bottom panel) cells were detected by immunofluorescence staining. Scale bars, 30 μm.

DUBs participate in a variety of cellular processes by cleaving the ubiquitin chains of substrates, increasing protein stability and increasing protein levels in cells. Here, to identify the substrate of USP12, the proteomic profile of USP12-overexpressing and control HEK293T cells was assessed by the isobaric tags for relative and absolute quantification (iTRAQ) technique (Fig. 3A). A total of 36 differentially expressed proteins (DEPs) (including 21 upregulated proteins and 15 downregulated proteins) were identified. Among them, MDK plays a key role in metastasis and angiogenesis [20, 21] and was upregulated by USP12 overexpression. We confirmed this result via western blotting and found that USP12 but not the USP12 mutant (C48A) upregulated the MDK protein level in HEK293T cells (Fig. 3B) but rarely affected the MDK mRNA level (Fig. 3C). To further clarify whether USP12 directly targets MDK, we assessed the interaction between USP12 and MDK in HEK293T cells after co-expression of USP12-Flag and MDK-GST. The co-immunoprecipitation (co-IP) results showed that USP12 could interact with MDK in cells (Fig. 3D, E). Moreover, our data also showed that the there was an interaction between endogenous MDK and USP12 (Fig. 3F). To further reveal the relationship between USP12 and MDK, we determined their cellular localization in MDA-MB-231 and MCF7 cells by immunofluorescence analysis. As expected, USP12 mainly co-localized with MDK in the cytoplasm (Fig. 3G). These results suggest that the protein interaction between USP12 and MDK could occur at the physiological level.

### USP12 stabilizes MDK through deubiquitination

**Fig. 4:**
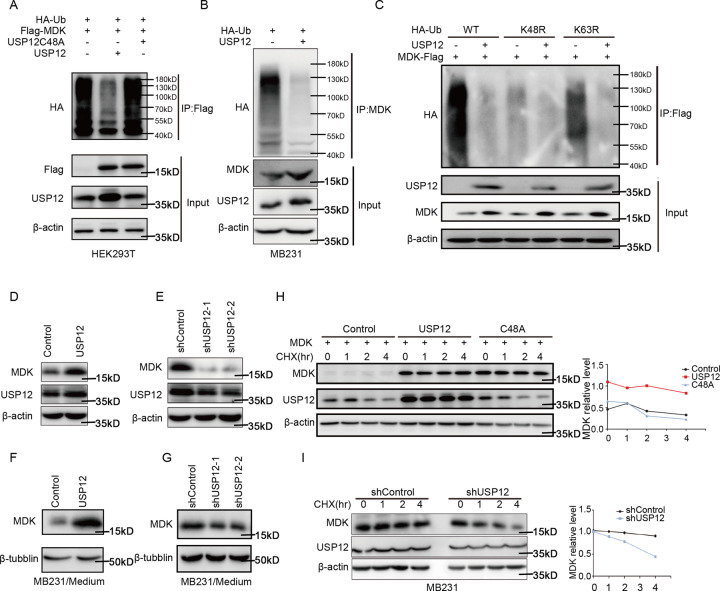
USP12 stabilized MDK through deubiquitination. **A** MDK-Flag and HA-UB plasmids with USP12, USP12 mutant (C48A) and control vector were co-transfected into HEK293T cells for 36 h. After 6 h of incubation with 10 μM MG132, ubiquitination assay was performed to detect the poly-ubiquitination of MDK. **B** The endogenous poly-ubiquitination level of MDK in MDA-MB-231 cells after USP12 overexpression was detected by the deubiquitination assay. **C** Indicated plasmids were transfected into HEK293T cells; 36 h after transfection, the cells were treated with MG132 for 6 h and then subjected to ubiquitination assay. **D** USP12 was stably overexpressed in MDA-MB-231. Immunoblotting showed the protein levels of MDK and USP12. **E** USP12 was knocked down in MDA-MB-231 cells. Immunoblotting showed the protein levels of MDK and USP12. **F** USP12 was overexpressed in MDA-MB-231 cells, and the MDK level in the medium supernatant of MDA-MB-231 cells was detected by immunoblotting. **G** USP12 was knocked down in MDA-MB-231 cells, and the MDK level in the medium supernatant of MDA-MB-231 cells was detected by immunoblotting. **H** MDK was co-expressed with USP12, USP12 mutant (C48A) and control vector in HEK293T cells. After 36 h, cells were treated with CHX (50 µg/ml) for the indicated time intervals. The expression of MDK and USP12 was detected (left panel), and the intensity of MDK expression was quantified by ImageJ software (right panel). **I** USP12 was knocked down with shRNA in MDA-MB-23 cells, and the MDK half-life was analysed by CHX pulse-chase assay with immunoblotting (left panel) and quantified (right panel).

Next, we determined whether USP12 deubiquitinates MDK proteins in cells. To this end, we co-expressed MDK-GST with USP12 and USP12 mutants (C48A) in HEK293T and MDA-MB-231 cells, and the ubiquitination results showed that MDK ubiquitination could be abolished by USP12 overexpression but not by overexpression of USP12 mutant (Fig. 4A). To further extend these findings, ubiquitination assay of endogenous MDK was also performed. We found that overexpression of USP12 could reduce the ubiquitination level of endogenous MDK in MDA-MB-231 (Fig. 4B) and MCF7 cells (Fig. S3A). To reveal the mechanism underlying maintenance of USP12 in MDK stability, we also identify the polyubiquitin-chains type of MDK deubiquitination by USP12. Here, wild-type ubiquitin or K48R mutant and K63R mutant ubiquitin were used to check the ability of USP12 to mediate different polyubiquitin chain types of MDK deubiquitination. As expected, we observed that K48R mutant, not K63R, markedly decreased the level of MDK ubiquitination and also reduced the effect of USP12 on deubiquitination of MDK protein (Fig. 4C). This result suggested that USP12 mediated MDK deubiquitination by cleaving K48, but not K63, linked polyubiquitin chains. In addition, overexpressing USP12 increased the endogenous MDK protein levels in MDA-MB-231 (Fig. 4D) and MCF7 cells (Fig. S3B), and knocking down USP12 decreased the endogenous MDK protein levels in MDA-MB-231 (Fig. 4E) and MCF7 cells (Fig. S3C). Moreover, overexpressing USP12 (Fig. 4F) also increased the MDK levels in the medium supernatant of MDA-MB-231 cells), and knocking down (Fig. 4G) USP12 decreased the MDK protein levels in the medium supernatant of MDA-MB-231 cells. To further validate whether MDK deubiquitination mediated by USP12 affects MDK protein stability, we transfected HEK293T cells with USP12 and USP12 mutants (C48A) and performed a cycloheximide (CHX) pulse-chase assay. The results showed that overexpression of USP12 remarkably increased the stability of the MDK protein compared with that in the control HEK293T cells, and there was no such effect with the USP12 mutant (Fig. 4H). Moreover, our data demonstrated that knocking down USP12 decreased endogenous MDK protein stability in MDA-MB-231 and MCF7 cells (Figs. 4I and S3D). These results suggest that USP12 regulates MDK expression by deubiquitinating and stabilizing MDK in cells.

### USP12 promotes angiogenesis in breast cancer by upregulating MDK

**Fig. 5:**
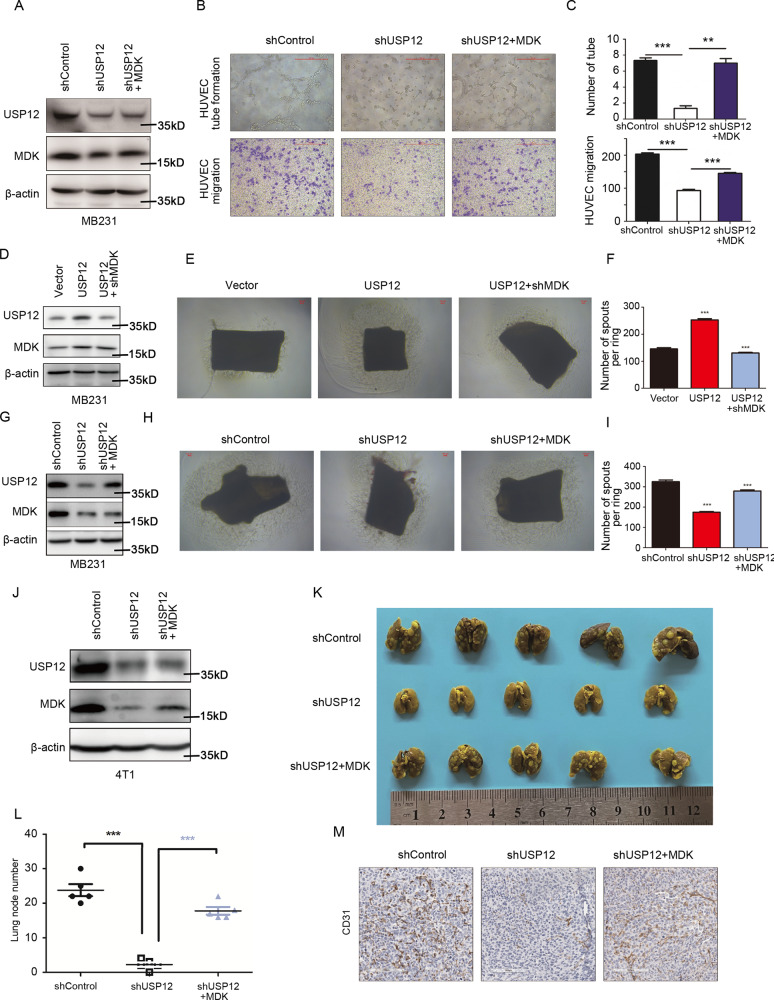
USP12 promoted angiogenesis in breast cancer by upregulating MDK. **A** MDK and control vectors were transfected into MDA-MB-231 cells after USP12 knockdown, and immunoblotting showed the protein levels of MDK and USP12. **B**, **C** The angiogenesis of HUVECs was analysed by tube formation and migration assays (**B**). The tubes and migratory cells in panel B were quantified (**C**). **D**–**I**. The MDK and USP12 expression were analysed by immunoblotting MDK was knocked down in MDA-MB-231 USP12-overexpression cells (**D**–**F**) or overexpressed in MDA-MB-231-shUSP12 cells (**G**–**I**). The angiogenesis was analysed by the mouse aortic ring assay (**E**, **G**). Scale bars, 100 µm. **J** MDK and control vector were transfected into 4T1 cells after USP12 knockdown, and the protein levels of MDK and USP12 were detected by immunoblotting. **K** Lung metastasis nodules 5 weeks after the injection of 4T1 cells with USP12 knockdown and MDK overexpression into mouse mammary pads; the number of nodules in (**K**) was calculated (**L**). The data are presented as the mean ± SD, ^***^p < 0.001. **M** CD31 immunohistochemical staining of lung metastatic nodules in (**K**) is shown.

To assess whether USP12 promotes breast cancer angiogenesis by upregulating MDK, we overexpressed MDK in MDA-MB-231 and MCF7 cells with USP12 knockdown **(**Figs. 5A and S4A) and measured angiogenesis by HUVEC migration and tube formation assays in vitro. Our rescue experiment results showed that MDK overexpression rescued the decreased HUVEC migration and tube formation ability mediated by USP12 depletion in MDA-MB-231 (Fig. 5B, C) and MCF7 cells (Fig. S4B, C). We further verify that USP12 induces breast cancer angiogenesis through MDK by using the mouse aortic ring assay. MDK gene was knocked down at the same time in breast cancer cells overexpressing USP12. MDA-MB-231 cell, and process the mouse aorta for 1 week by the cell supernatant. We found that the effect of USP12 in promoting angiogenesis was significantly reversed by the deletion of MDK (Fig. 5D–F). Conversely, ectopic expression of MDK in MDA-MB231-shUSP12 cells obviously induces neovascularization in the mouse aortic ring and almost neutralized the function of the knockdown of USP12 (Fig. 5G–I). In addition, MDK can activate the Akt signalling pathway in tumour and endothelial cells and promote VEGFR3 expression through mTOR signalling pathway to promote tumorigenesis and development [20]. Here, we checked the level of Phospho-Akt in breast cancer MDA-MB-231 cells after knocking down USP12, and we found that knocking down of USP12 can reduce the Phospho-Akt (Ser473) level (Fig. S5A). Furthermore, we detected the expression of VEGFR3 in endothelial cells treated with breast cancer cell supernatant, and found that the supernatants of MB-MDA-231 cells knockdown USP12 and this inhibits the expression of VEGFR3 protein in endothelial cells (Fig. S5B). Collectively, these results indicate that USP12 could promote angiogenesis in breast cancer through upregulating MDK. We further confirmed this result in the orthotopic mouse lung metastasis model, and the results showed that USP12 depletion in 4T1 cells decreased the lung metastasis ability and CD31 (a vascular endothelial cell marker) protein level of lung metastatic nodules in orthotopic mice, whereas overexpression of MDK attenuated the reduction in lung metastasis ability (Fig. 5J–L) and CD31 protein expression of lung metastatic nodules in 4T1 cells mediated by USP12 depletion (Fig. 5M). These results demonstrated that USP12 promotes breast cancer angiogenesis and metastasis by upregulating MDK.

### High USP12 and MDK expression predicts a poor prognosis in breast cancer patients

**Fig. 6:**
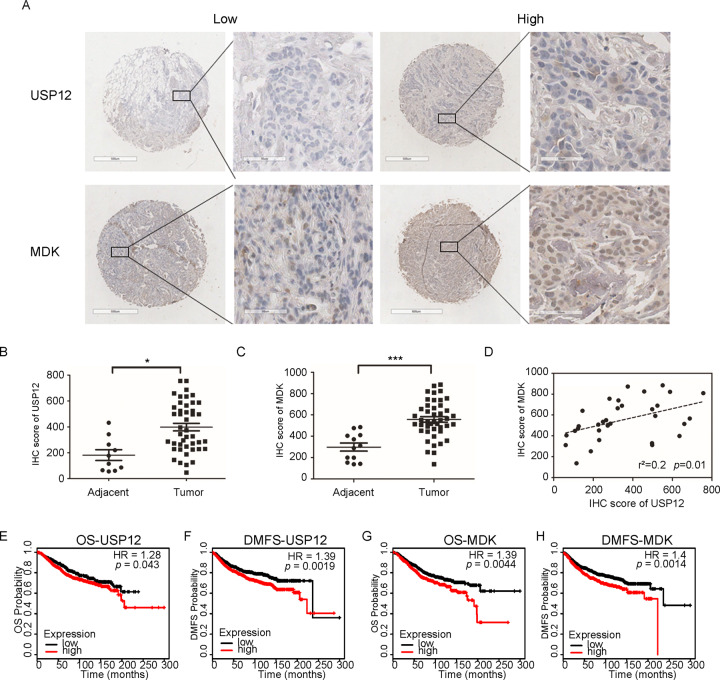
High USP12 and MDK expression predicts a poor prognosis in breast cancer patients. **A** Representative staining of USP12 and MDK in breast cancer samples. Scale bars, 100 μm. **B**, **C** Comparison of the relative protein expression levels of USP12 and MDK in 32 paired breast cancer and adjacent normal tissues. ^*^p < 0.05, ^***^p < 0.001; mean ± SD. **D** A positive correlation was observed between USP12 and MDK protein expression in breast cancer samples. **E–H** The relationship between USP12 or MDK expression and the OS or DMFS rate of breast cancer patients was analysed in the KM (http://kmplot.com/analysis/) database.

To further validate the clinical significance of USP12 and MDK in breast cancer metastasis, we detected their expression levels in breast cancer clinical tissue specimens (Fig. 6A). Our data revealed that USP12 and MDK were both highly expressed in breast cancer tissues compared with adjacent tissues (Fig. 6B, C). Moreover, the USP12 level was positively correlated with the MDK level in breast cancer tissue samples (Fig. 6D). Finally, we also analysed the relationship between USP12 and MDK expression and the prognosis of breast cancer patients from the KM (http://kmplot.com/analysis/) database. The data showed that the patients with high USP12 expression had poorer overall survival (OS) rates (*p* = 0.043) and DMFS rates (*p* = 0.0019) than those with low USP12 levels (Fig. 6E, F). Consistently, the patients with high MDK expression also had poorer OS rates (*p* = 0.0044) and DMFS rates (*p* = 0.0014) than those with low MDK levels (Fig. 6G, H).

## Discussion

In this study, through screening DUBs related to breast cancer DMFS in the KM Plotter database and confirming the results with the in vivo metastasis mouse model, we identified that USP12 could be a chief contributor to breast cancer metastasis. KM survival analysis showed that the DMFS of breast cancer patients with high USP12 levels was significantly worse than that of patients with low USP12 levels. Moreover, an in vivo mouse model showed that USP12 knockdown remarkably decreased the metastatic ability of 4T1 cells, while USP12 overexpression increased the metastatic ability of 4T1 cells. Angiogenesis, that is, the formation of new blood vessels, is an essential component in tumour growth and haematogenous metastasis, and vascular density can be a prognostic indicator of metastatic potential [22]. Here, we demonstrated that USP12 increased metastasis by promoting angiogenesis. First, we found that the supernatant from USP12-overexpressing MDA-MB-231 cells promoted the migration of HUVECs and that the supernatant from USP12-knockdown MDA-MB-231 cells significantly decreased HUVEC migration. Second, tube formation assays demonstrated that compared with that from control cells, the supernatant from USP12-overexpressing MDA-MB-231 cells obviously promoted HUVEC angiogenesis. Moreover, overexpression of USP12 promoted the secretion of VEGF-A and VEGF-C, and knockdown of USP12 inhibited the secretion of these factors.

DUBs participate in a variety of cellular processes by increasing the stability and protein levels of their substrates in cells [3, 4]. To further clarify the mechanism by which USP12 promotes metastasis, iTRAQ combined with LC-MS/MS was performed to identify the DEPs in USP12-overexpressing cells. Our results showed that USP12 remarkably increased MDK protein expression compared with that in the control cells. MDK is a small exosomal molecule that has been reported to be expressed in a variety of tumours and is associated with angiogenesis [21, 23–25]. The secretion of MDK by melanoma cells affects the expression of VEGFR3 on lymphatic endothelial cells through the paracrine effect of the mTOR pathway [21]. It is abnormally expressed at high levels in various cancers [18], especially during tumour progression into more advanced stages [19]. Our cellular immunofluorescence results showed that the USP12 and MDK proteins mainly co-localized in the cytoplasm. To clarify the mechanism by which USP12 regulates MDK protein expression, we examined the interaction between USP12 and MDK. As expected, our results demonstrated that USP12 could directly interact with MDK, decrease its polyubiquitination and increase its protein stability in cells. USP12 also promoted MDK protein expression in MDA-MB-231 extracellular medium. Moreover, coordinated upregulation of USP12 and MDK was demonstrated in clinical breast cancer samples. A strong positive relationship between the protein level of USP12 and MDK was found in these samples, which confirmed the regulatory relationship between these two proteins.

Furthermore, our data revealed that USP12 promoted breast cancer metastasis by upregulating MDK protein levels. Consistent with USP12, high MDK expression also predicted a poor prognosis in breast cancer patients. Ectopic expression of MDK markedly promoted angiogenesis and almost neutralized the effects of USP12 knockdown in MCF7 and MDA-MB-231 cells. USP12 depletion dramatically decreased the number of lung metastatic nodules, and overexpression of MDK attenuated the effect in vivo.

Collectively, our study revealed that USP12 is responsible for deubiquitinating and stabilizing MDK, which induces breast cancer metastasis by promoting angiogenesis, and provides a novel regulatory mechanism for breast cancer metastasis. Therefore, the USP12–MDK axis could serve as a potential target for the therapeutic treatment of breast cancer metastasis.

## Materials and methods

### Cell culture and transfection

The MDA-MB-231, MCF7, 4T1 and HEK293T cell lines were obtained from the American Type Culture Collection (ATCC). MCF7 cells were maintained in DMEM (HyClone, USA) supplemented with 10% fetal bovine serum, 2 mM glutamine, 0.01 mg/ml insulin and 1% penicillin/streptomycin, and MDA-MB-231 cells were maintained in L15 medium (HyClone, USA) supplemented with 10% fetal bovine serum. HUVECs were obtained from the China Infrastructure of Cell Line Resource. 4T1, HEK293 and HUVECs were maintained in DMEM (HyClone, USA) supplemented with 10% fetal bovine serum. Plasmid transfection was performed using Lipofectamine 2000 (Invitrogen, USA) according to the manufacturer’s instructions.

### Plasmid construction

The shRNA sequences were cloned into the pSIH1 lentiviral shRNA expression vector. The lentiviruses encoding shRNA sequences for targeting genes were produced according to the manufacturer’s instructions. The targeting sequences of genes were as follows:

ATXN3: ACGAGAAGCCTACTTTGAA; BAP1: GAGCAAAGGATATGCAATT; JOSD1: CCACAAATCTACCATGAGA; OTUD4: GGGTAGGACAAGTGGAAAT; PSMD14: GTGCTTATGACTTCAAATA; PSMD7: GCAAAAGAAAGTACTTGATGT; UCHL1: CCGAGATGCTGAACAAAGT; USP1: CACAGTGGCATTACTATTA; USP4: CCAAATGGATGAAGGTTTA; USP5: GGACAACCCTGCTCGGATC; USP9X: GTTCGAAGATGTATACTCA; USP11: GAACAAGGTTGGCCATTTT; USP12: GAAACTCTGTGCAGTGAAT; USP14: GGAATTGCCATGTGGATTG; USP12 (#1): CCAGATGTCTTACTTGTGAAA; and USP12 (#2): CCTTTAGAACTTCGTCTGTTT.

4T1 cells were infected with these lentiviruses, and stable knockdown cells were obtained following selection with 1 μg/ml puromycin for 1 week and knockdown effect was verified by qPCR (Table S1). The MDK, USP12 and USP12 mutant (C48A) were cloned into the pLVX-IRES-puro vector with or without specific tags and verified by DNA sequencing.

### Antibodies and reagents

The antibodies used for Western blotting and IP in this study included anti-MDK antibody (sc-46701, Santa Cruz, USA), anti-USP12 antibody (LS-C370534, LSBio, USA), anti-CD31 antibody (77699, CST, USA), anti-Flag antibody (14793, CST, USA), anti-GST antibody (2624, CST, USA), anti-HA antibody (3724, CST, USA), mouse IgG (3420, CST), anti-GST agarose antibody (A8580, Sigma Aldrich) and anti-Flag^®^ M2 affinity gel (A2220, Sigma Aldrich). MG132 (C2211, Sigma Aldrich) was dissolved in dimethyl sulfoxide. Matrigel matrix (356234, Corning), the human VEGF-A ELISA kit (EKO588-96, Boster) and the human VEGF-C ELISA KIT (EKO539-96, Boster) were also used.

### Migration and invasion assay

Transwell invasion and migration assays were performed according to the manufacturer’s instructions. Cells were suspended in serum-free medium and seeded into Transwell chambers. The bottom chamber was filled with medium with 10% FBS. After 24 h, the invasive cells were stained with crystal violent and counted. The migration experiment was similar to the invasion experiment, without Matrigel coating in the upper chamber.

### Angiogenesis assay

Herein, angiogenesis was measured via HUVEC migration assays, tube formation assays and angiogenesis marker (such as VEGF and CD31) assessment. For the HUVEC migration assay, HUVECs treated with breast cancer cell (MDA-MB-231 and MCF7) supernatant were suspended in serum-free medium and seeded into Transwell chambers. The bottom chamber was filled with medium with 10% FBS and breast cancer cell supernatant. After 24 h, the invasive cells were stained with crystal violet and counted. For the tube formation assay, 96-well plates were coated with Matrigel and incubated at 37 °C for 30 min. HUVECs treated with supernatant for 48 h were cultured for 6 h with the collected breast cancer cell supernatant in a 96-well plate, covered with Matrigel, and then photographed under a microscope. Tube formation ability was determined by counting the number of tubes.

In addition, the angiogenesis markers VEGF-A and VEGF-C in the supernatant medium of different cells after various treatments were detected by ELISA using a human VEGF ELISA kit and human VEGF-C ELISA kit in accordance with the manufacturer’s instructions. The vascular endothelial cell marker CD31 was also assessed by IHC for angiogenesis analysis in a mouse model.

### Immunofluorescence assay

Cells grown on glass coverslips were fixed in 4% paraformaldehyde for 20 min at room temperature. The cells were washed three times with PBS. Blocking buffer 5% BSA was added for 1 h. The cells were then incubated with anti-USP12 and anti-MDK primary antibodies overnight, with secondary antibodies conjugated with Alexa Fluor in a darkroom for 1 h, and then with 0.5 μg/ml DAPI for 5 min. The cells were visualized with a laser confocal microscope.

### Immunoprecipitation (IP) assay

After the HEK293T cells were transfected with the plasmids for 48 h, they were collected, and IP protein lysis buffer was added. After the cell lysate was incubated on ice for 45 min, the sample was centrifuged at 12,000*g* for 10 min, and the supernatant was collected. A small amount was taken as input, and the others were incubated with anti-Flag M2 affinity gel overnight at 4 °C. The next day, the sample was centrifuged at 3000*g* for 5 min at 4 °C. We discarded the supernatant and washed the beads with PBS three times. Then, we added 15 μl of 2× SDS loading buffer to the pellet and boiled it for 5 min. Finally, the IP protein was detected by western blot analysis.

### Deubiquitination assay

USP12 or USP12-C48A, MDK-flag and HA-UB were co-transfected into HEK293T cells, and MG132 was added 6 h before collecting the cells. Then, the cell pellet was treated with IP protein lysate and incubated on ice for 45 min. After that, the samples were centrifuged at 12,000*g* and 4 °C for 10 min. We collected the supernatant and used a part as input, and the remaining part was incubated with anti-Flag M2 affinity gel at 4 °C overnight. The next day, those samples were centrifuged at 3000*g* at 4 °C for 5 min. After we washed the beads three times with PBS, we added 15 μl of 2× loading buffer to the samples and then boiled them for 5 min.

### Orthotopic mouse lung metastasis model

A total of 3 × 10^5^ 4T1 cells were injected into the mammary pads of 6-week-old female BALB/c mice (Vital River, Beijing, China). Ten mice were randomly selected for each group. Six weeks after injection, the lungs were harvested and stained with picric acid, and the number of lung metastatic nodules was counted. The experimental procedures were approved by the Institutional Animal Care and Use Committee of Cancer Hospital Chinese Academy of Medical Sciences. ^∗∗^*p* < 0.01 by Mann-Whitney *U* test.

### Immunohistochemistry assay

Breast cancer tissue arrays were obtained from the Cancer Hospital Chinese Academy of Medical Sciences. The experimental process was approved by the ethical committee of the hospital. Immunohistochemistry (IHC) was performed with primary antibodies against USP12 and MDK according to a standard protocol and the manufacturer’s instructions. We scanned the arrays using the Aperio ImageScope system (Leica Biosystems, USA). Semiquantitative analysis of USP12 and MDK protein levels performed using ImageScope software (Aperio Technologies) was described in a previous study [26].

### Proteomic profile of cells detected by the iTRAQ technique

First, the cells were sonicated three times on ice using a high-intensity ultrasonic processor (Scientz) in lysis buffer (8 M urea, 1% Protease Inhibitor Cocktail). Then, the protein samples were centrifuged at 12,000 rpm for 10 min at 4 °C. Finally, the supernatants were collected. Protein preparation and LC-MS/MS detection using the iTRAQ technique were performed at Jingjie PTM Biolab Co., Ltd. (Hangzhou, China).

A total of 6816 proteins (<1% FDR) were identified in the USP12-overexpressing and control HEK293T cells, and 6100 proteins had related quantitative information (Table S2). The proteins that met the following criteria were considered DEPs: an average ratio-fold change >1.25 or <1/1.25 and the same change trend in the three experiments between the two cells (*t*-test; *p* < 0.05). In total, 36 DEPs, including 21 upregulated proteins and 15 downregulated proteins, were identified.

### Statistical analysis

To assess the correlation between USP12 and MDK expression in cancer specimens, statistical analysis was performed using Pearson’s test with SPSS software version 22.0. All data followed a normal distribution, and no sample was excluded. For other statistical analyses, one-way ANOVA or Student’s *t*-test was used with GraphPad Prism Version 5.01 software. Data are presented as the mean ± SEM of independent experiments. Differences with a two-tailed *p* value < 0.05 were considered statistically significant.

## Supplementary information

Supplementary legends.

Supplemental Fig. 1.

Supplemental Fig. 2.

Supplemental Fig. 3.

Supplemental Fig. 4.

Supplemental Fig. 5.

Table S1.

Table S2.

Authors’ contribution.

Checklist.
