# Supplementary material for: USP12 promotes breast cancer angiogenesis by maintaining midkine stability
Source: Cell Death Dis. 2021 Nov 11;12(11):1074. doi: 10.1038/s41419-021-04102-y (PMC8580968; doi:10.1038/s41419-021-04102-y)
Supplement: Supplementary file 1 — Supplementary legends. [file 41419_2021_4102_MOESM1_ESM.docx]

**Supplemental Figure**

**Fig. S1.** USP12 induces angiogenesis in breast cancer. A. Migration and invasion assays were performed using USP12 stable overexpression MDA-MB-231 and control cells (top), and the quantitative results are shown (bottom). B. Migration and invasion assays were performed using stable USP12 knockdown and control MDA-MB-231 cells (top). The quantitative results are shown (bottom). C. Tube formation assay and migration of HUVECs treated with supernatant from MCF7/vector and MCF7/USP12 cells (top), and representative images are shown (bottom). The quantitative results are shown in the bottom panel. D. Tube formation and migration assays of HUVECs treated with supernatant from MCF7/shControl, MCF7/shUSP12-1 and MCF7/shUSP12-1 cells. Representative images (top) and quantitative results (bottom) are shown. The experiments were repeated three times, and the results from one experiment are shown. ***p < 0.001, mean ± SD.

**Fig. S2**. USP12 stabilizes MDK through deubiquitination. A. The poly-ubiquitination level of MDK in USP12 knockdown MCF7 cells was detected by a deubiquitination assay. B. USP12 was overexpressed in MCF7 cell. Immunoblotting showed the protein levels of MDK and USP12. C. USP12 was knocked down in MCF7 cells. Immunoblotting showed the protein levels of MDK and USP12. D. USP12 was knocked down with shRNA in MCF7 cells. The MDK half-life was analysed by CHX pulse-chase assay (left panel), and the quantitative results are shown (right panel).

**Fig. S3**. USP12 promotes MDK-induced angiogenesis in breast cancer. A. USP12 was stably knocked down in MCF7 cells, and MDK was overexpressed in MCF7-shUSP12 cells. Immunoblotting showed the protein levels of MDK and USP12. B. The angiogenesis of HUVECs was analysed by tube formation assay (top) and migration assay (bottom). C. The migratory cells (left) and tubes (right) in B were counted.
