# Supplementary figures and images for: USP12 promotes breast cancer angiogenesis by maintaining midkine stability
Source: Cell Death Dis. 2021 Nov 11;12(11):1074. doi: 10.1038/s41419-021-04102-y (PMC8580968; doi:10.1038/s41419-021-04102-y)

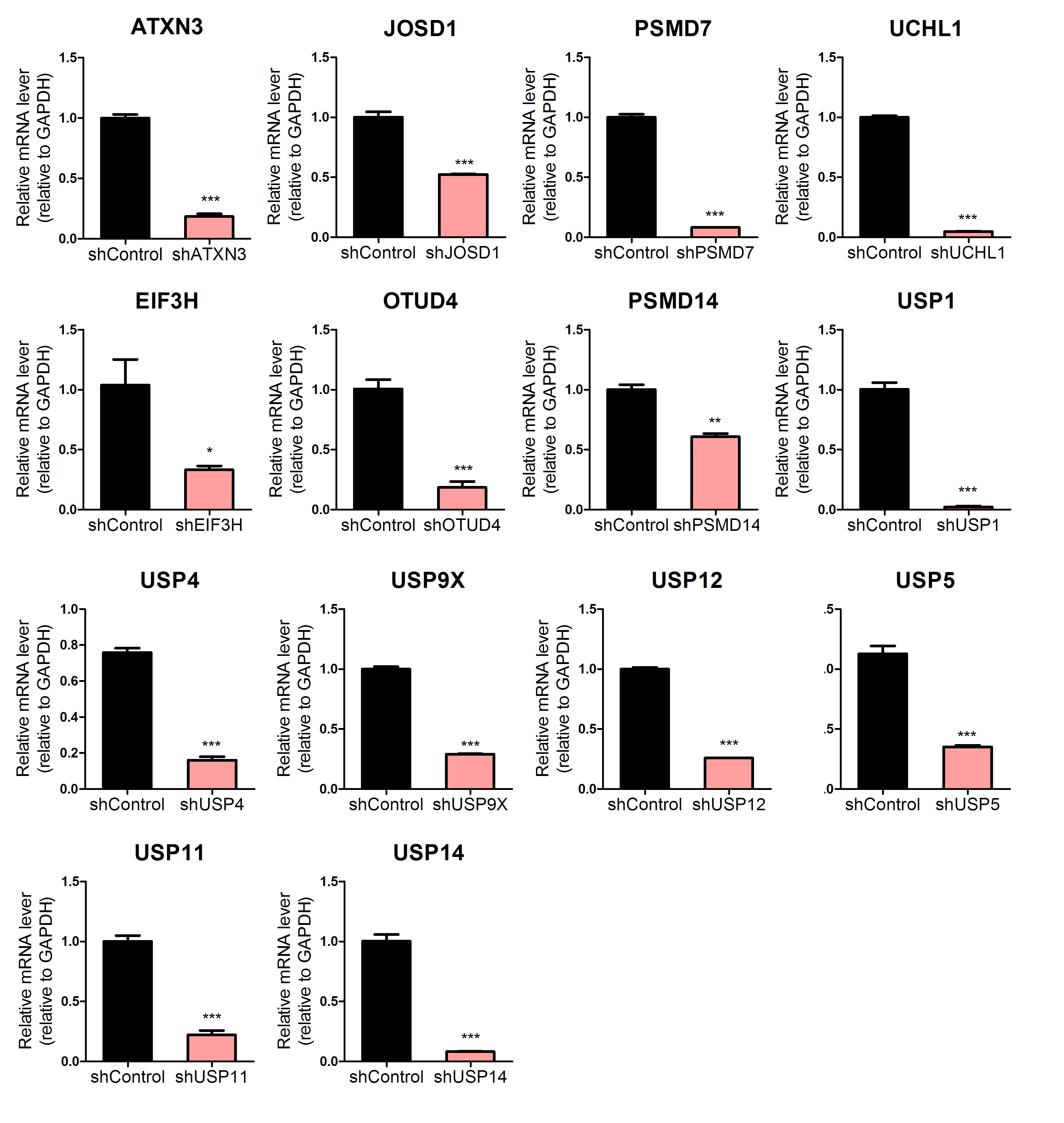

Supplement: Supplementary file 2 — Supplemental Fig. 1. [file 41419_2021_4102_MOESM2_ESM.tif]

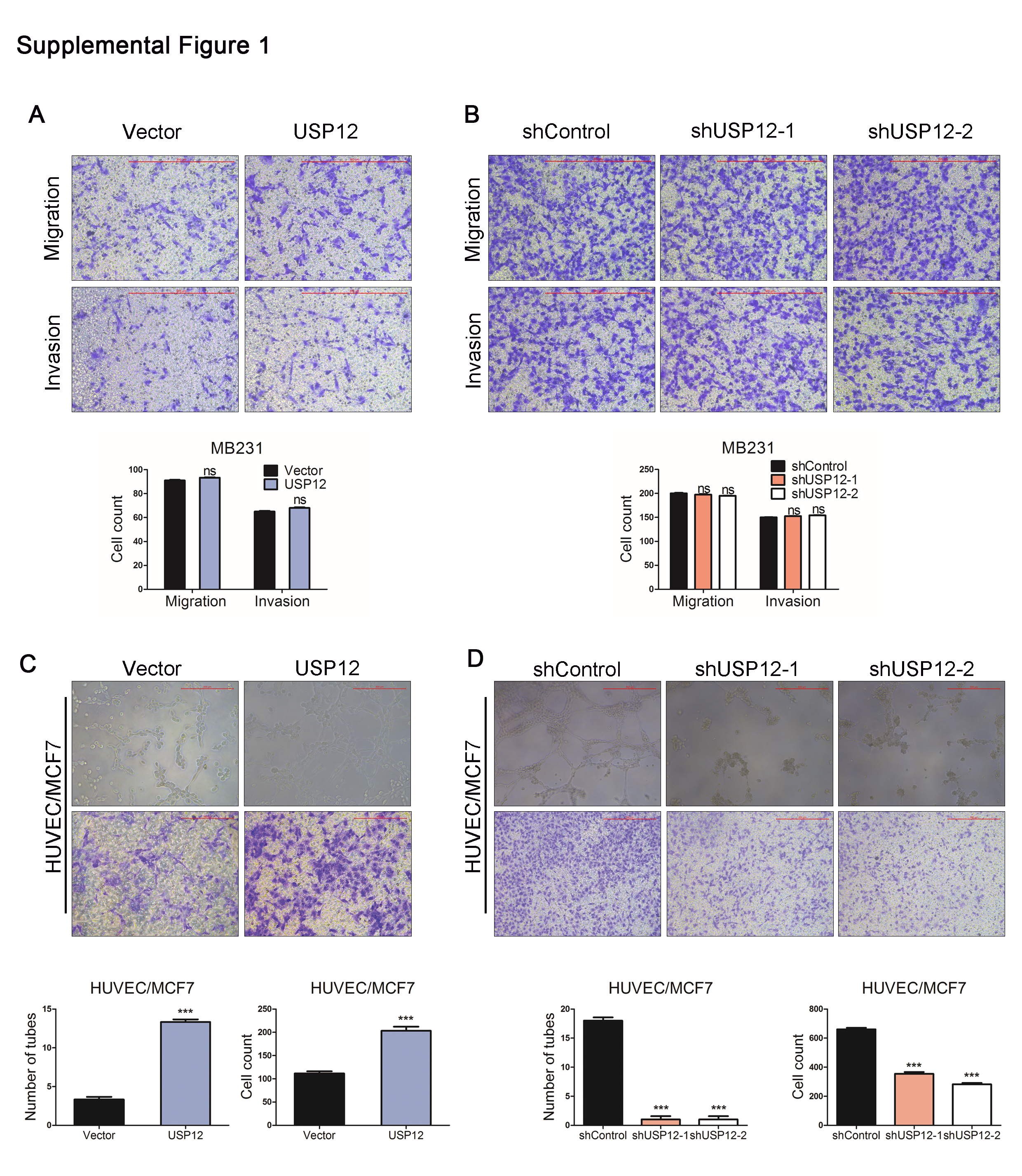

Supplement: Supplementary file 3 — Supplemental Fig. 2. [file 41419_2021_4102_MOESM3_ESM.tif]

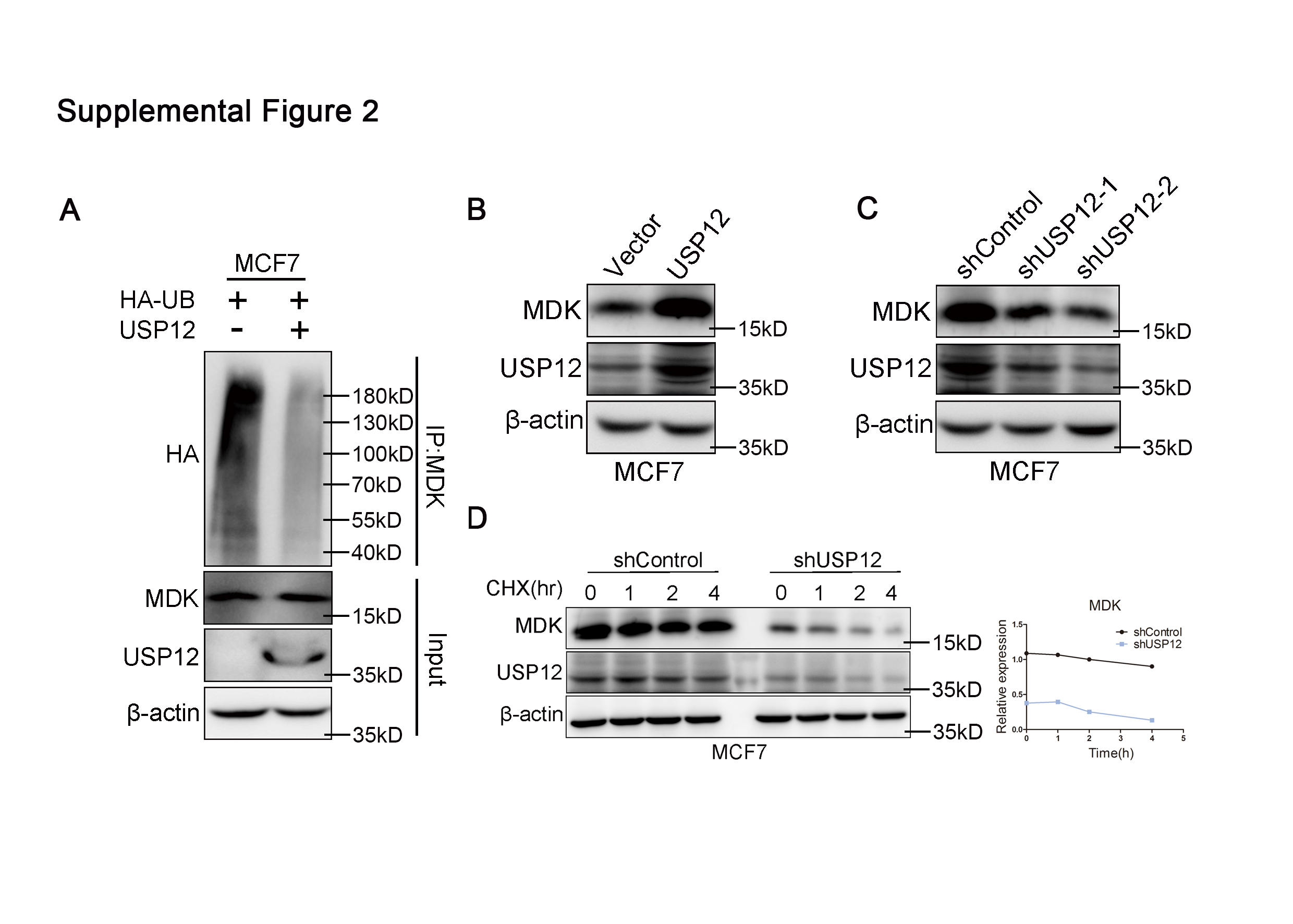

Supplement: Supplementary file 4 — Supplemental Fig. 3. [file 41419_2021_4102_MOESM4_ESM.tif]

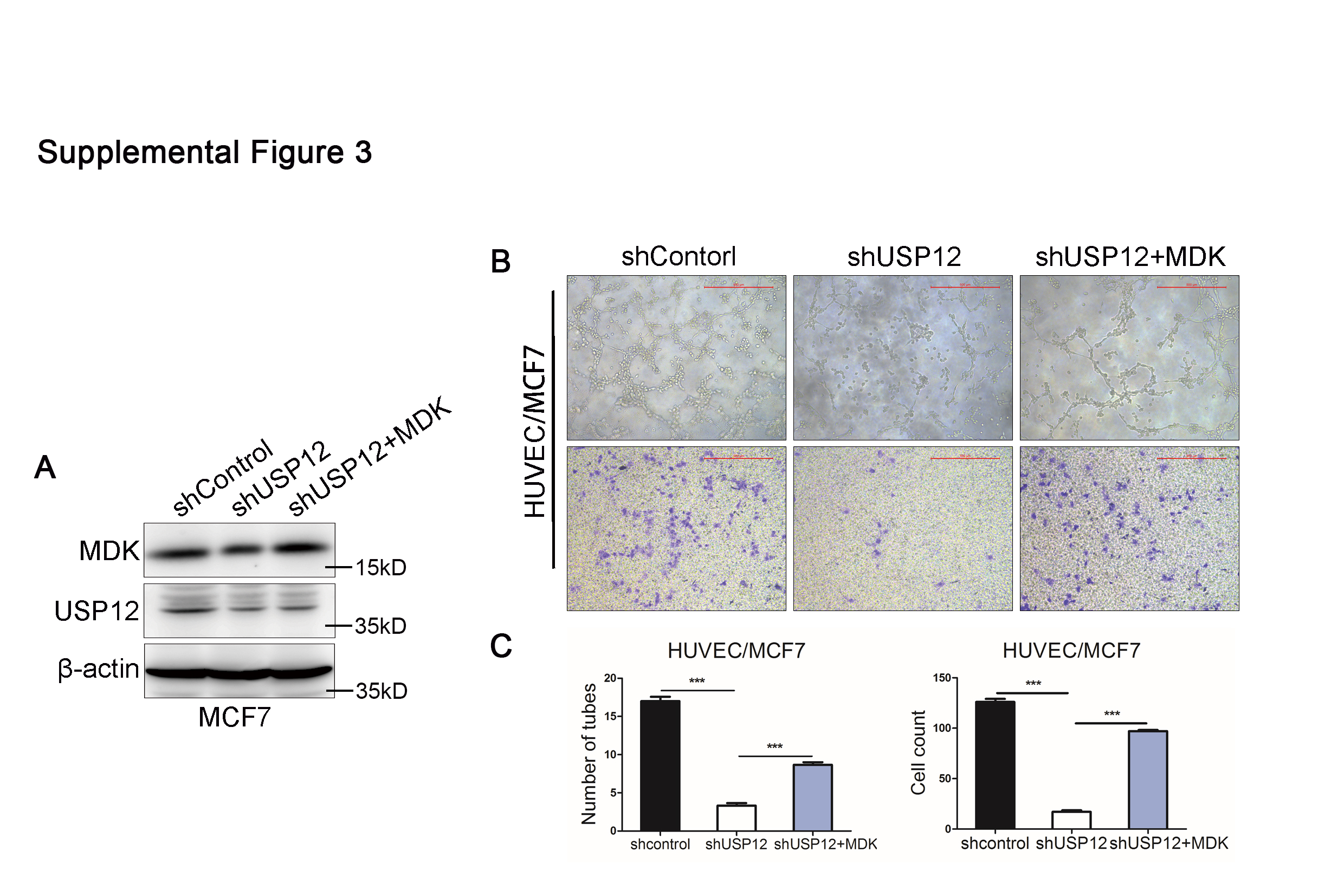

Supplement: Supplementary file 5 — Supplemental Fig. 4. [file 41419_2021_4102_MOESM5_ESM.tif]

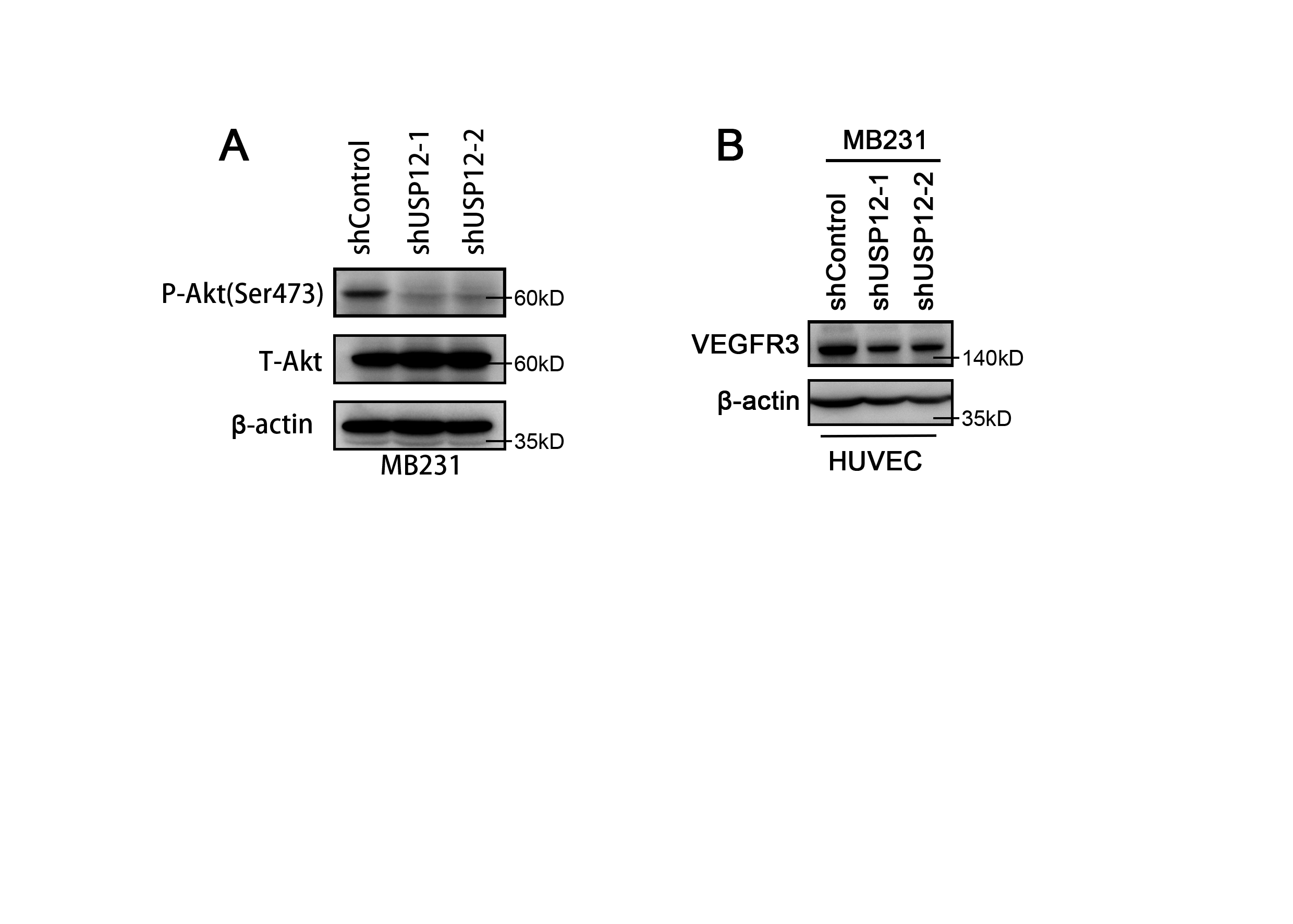

Supplement: Supplementary file 6 — Supplemental Fig. 5. [file 41419_2021_4102_MOESM6_ESM.tif]
