# Supplementary material for: USP12 promotes breast cancer angiogenesis by maintaining midkine stability
Source: Cell Death Dis. 2021 Nov 11;12(11):1074. doi: 10.1038/s41419-021-04102-y (PMC8580968; doi:10.1038/s41419-021-04102-y)
Supplement: Supplementary file 9 — Authors’ contribution. [file 41419_2021_4102_MOESM9_ESM.pdf]

DECLARATION OF CONTRIBUTIONS TO ARTICLE **ADMC**

Manuscript Number:	Journal Name:	
CDDIS-21-1019R	*Cell Death & Disease*	(the 'Journal')

Proposed Title of the Contribution:

Authors' contribution table (the 'Contribution)

Author(s):

Bin Sheng; Zichao Wei; Xiaowei Wu; Yi Li; Zhihua Liu (the 'Authors')

For all *CDDis* articles, each person named as an author in the published version must be able to show he or she has contributed substantially to the article.

Authorship credit should be based on 1) substantial contributions to conception and design, acquisition of data, or analysis and interpretation of data; 2) drafting the article or revising it critically for important intellectual content; and 3) final approval of the version to be published. Authors should meet conditions 1, 2 and 3.

Any person who cannot be shown to have made a substantial contribution to the article cannot be listed as an author in the final version. The name of any person who is deemed to have made a minor contribution can, however, appear in the Acknowledgments section of the article.

Please complete the table below to indicate the contributions of all named authors to the manuscript.

Author Full Name:	Specification of Contribution to the Manuscript:
Bin Sheng	designed and performed experiments and write the manuscript
Zichao Wei	helped perform experiments
Xiaowei Wu	constructed plasmids and helped completed DUBs screening
Yi Li	designed experiments and helped write the manuscript.
Zhihua Liu	designed experiments and provided financial support

Please complete the table below to indicate the contributions of all named authors to the figures.

Figure 1:

Bin Sheng generated the data and assembled the figure with Yi Li;
Xiaowei Wu helped generate the results of Figures 1A and B

Figure 2:

Bin Sheng assembled the figure and generated the data with Zichao Wei

Figure 3:

Bin Sheng generated the data and assembled the figure with Yi Li;

Figure 4:

Bin Sheng generated the data and assembled the figure with Yi Li;

Figure 5:

Bin Sheng assembled the figure and generated the data with Zichao Wei

Figure 6:

Bin Sheng generated the data and assembled the figure with Yi Li;

Signed for and on behalf of the Author(s):	Print Name:	Date:
[signature]	Zhihua Liu	2021.7.7
